# Supplementary material for: Point of care lung ultrasound is useful when screening for CoVid-19 in Emergency Department patients
Source: medRxiv. 2020 Jun 12:2020.06.09.20123836. Preprint. [Version 1] doi: 10.1101/2020.06.09.20123836 (PMC7310645; doi:10.1101/2020.06.09.20123836)
Supplement: Supplement 2020 [file 85442-2020.06.09.20123836-1.docx]

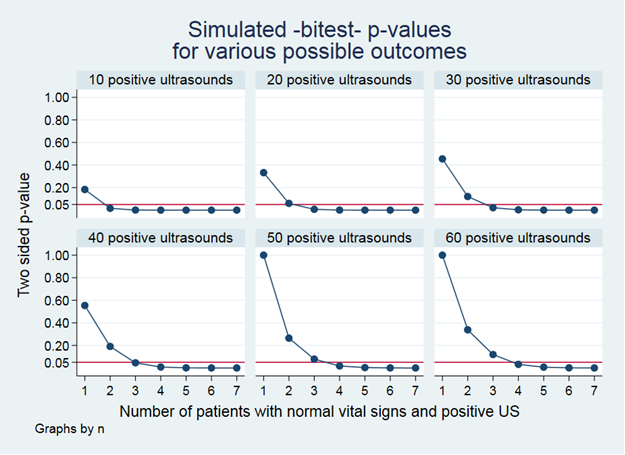


**Appendix 1. Sample size calculation**

Binomial probability testing using the bitest function in Stata statistical software.

Before undertaking the study we calculated p-values to test null P0=0.02 for plausible anticipated data

Code used to determine if likely sample sizes needed were plausible.

sample_size_simulator -

Page 1

1 //

2 //

3 // US in CoVid 19 detection

4 // Simulation of bitest to test the the null hypothesis that no

5 // Sacramento 4-16-20 Paul WALSH

6 //

7 frame change default

8

9 cap frame drop test

10

11 frame create test n obs_K exp_k assum_p obs_p p_val

12

13 forval x= 10(10)60 {

14

15 forval y=1/7 {

16

17 bitesti `x' `y' 0.02 ,detail

18

19 frame post test (r(N)) (r(k)) ( r(P_p) *r(N)) (r(P_p)) (r(k)/r(N)) (r

(p))

20

21 }

22

23 }

24

25 frame test: save sim_test ,replace

26

27 frame change test

28

29

30 //

31

32 format p_val %03.2f

33

34 lab var p_val "Two sided p-value"

35 lab var obs_K "Number of patients with normal vital signs and positive US"

36

37 cap lab drop number

38 lab def number 10 "10 positive ultrasounds" 20 "20 positive ultrasounds" 30

"30 positive ultrasounds" 40 "40 positive ultrasounds" 50 "50 positive

ultrasounds" 60 "60 positive ultrasounds"

39 lab val n number

40

41 tw (connect p_val obs_K , by(n ,title("Simulated -bitest- p-values" "for

various possible outcomes")) xlab(1/7) ylab(0.05 0.2 0.4 0.6 0.8 1) yline

(0.05) ylab(,angle(horizontal)) )

42

43 gr save simulations_nht ,replace

44

45

46
