# Supplementary material for: Point of care lung ultrasound is useful when screening for CoVid-19 in Emergency Department patients
Source: medRxiv. 2020 Jun 12:2020.06.09.20123836. Preprint. [Version 1] doi: 10.1101/2020.06.09.20123836 (PMC7310645; doi:10.1101/2020.06.09.20123836)
Supplement: Supplement 2020 [file 85442-2020.06.09.20123836-3.pdf]

```
//
//
/* This do file
    1. imports imaging reads and clinical findings that have previously been manually extracted into database elements (.mer file)

    2. Merges .dta file that contains the Vital signs and demographic data that can easily be done from EPIC

    3. Derives the variables needed for and performs the primary analysis

    4. Derives the Variables needed for Table 1.



Two other dof files will be needed.
    One to convert the raw textfile for 2 above into stata format.
    Second one to convert virus results data into wide format that will be needed for Table 1.
*/




//Safe harbor
// This will re assign all csn and acc and mrn new number
//    This is then used to populate a HIPAA safe database
//    that can used for further analysis
// End safe harbor
//


////Get datasets ready to merge
//vital signs data
clear
use "vitals_wide_CoVid_combined.dta"
isid csn
isid pud_id
save "H:\Covid\rough_work_merge2.dta" ,replace

// US data
//
clear
import delimited "H:\Covid\covid_US_second_pull.mer", clear
drop if csn =""
duplicates drop csn ,force
di _N
merge 1:1  csn using H:\Covid\rough_work_merge2.dta
keep if _merge  ==3
drop if regexm(acc, "under 2")
drop if regexm(acc, "duplicate")
list csn if  md_covid =""


// FOR CASES WHERE POCUS INTERP NOT DOCUMENTED IN MEDICAL RECORD
//  USE OVER READ INTERPRETATION

replace md_covid="Yes" if pud_id == pud_id==41
```

```
56  replace md_covid="No" if pud_id ==  pud_id==31
57  replace md_covid="No" if pud_id ==   pud_id==21
58
59
60
61
62  // Convert all yes to 1 and No to zero
63
64  /*
65  cap lab drop   yes_no
66  lab def yes_no 0 "No" 1 "Yes"
67
68  foreach var of varlist excess_long_b excess_short_b thickened_pleura
    effusion consolidation atelectasiss normal air_bronchograms
    excess_long_bcopy excess_short_bcopy thickened_pleuracopy effusioncopy
    consolidationcopy atelectasisscopy normalcopy air_bronchogramscopy
    md_covid ct_covid cxr_covid ct_ground_glass ct_bilateral ct_effusion
    ct_interst ct_lobar ct_peripheral ct_multifocal cxr_mulitfocal
    cxr_bilateral cxr_effusion cxr_lobar cxr_nil_acute_pulmn ct_nil_acut_pulm
    cough dyspnea subj_fever meas_fever   antipyr_home  ausc_clear
    ausc_crackles aus_wheez_ronc hx_chf hx_other_lung cw_covid
    not_being_screened_for_covid sore_throat fatigue headache myalgias
    diarrhea vomiting{
69
70    encode  `var' ,gen (`var'_bin)  lab( yes_no)
71    drop `var'
72  }
73
74  rename cw_covid_bin cw_covid
75
76
77  foreach var of varlist excess_long_bcopy_bin excess_short_bcopy_bin
    thickened_pleuracopy_bin  effusioncopy_bin  consolidationcopy_bin
    atelectasisscopy_bin normalcopy_bin  air_bronchogramscopy{
78
79  rename `var' mdUS_`var'
80
81  }
82
83  // For US over reads accept documentation by exception
84  foreach var of varlist excess_long_b_bin excess_short_b_bin
    thickened_pleura_bin effusion_bin consolidation_bin atelectasiss_bin {
85
86      replace `var' =0 if `var' ==.
87
88  }
89
90  assert normal_bin <.
91  assert normal_bin !=1 if (excess_long_b_bin + excess_short_b_bin
    +thickened_pleura_bin + effusion_bin + consolidation_bin +
    atelectasiss_bin) >0
92  assert cw_covid ==0 if normal_bin==1
93
94
95  */
96
97
98  // merge virus results file
```

```
99   //  drop _merge from previous merge after making sure it is correct
100  // Virus file will need ot be converted from long to wide
101
102
103  //Calculate age
104
105  lab var age "Age in years"
106
107
108  // Sort out gender
109
110
111  //sort out height - depends on units it arrives in assume inches for now
112  cap drop ht
113  gen ht =(height *2.54)/100
114  lab var ht "height in metres"
115  drop height
116
117  //sort out weight depends on the units it arrives in
118  destring weight,replace
119  replace weight =(weight/35.274)
120  lab var weight "Weight(Kg)"
121
122  //calculatete BMI
123  cap drop bmi
124  gen bmi = weight/(ht*ht)
125  lab var bmi  "BMI"
126  ////////////////////////
127
128
129  //Define as symptomatic
130
131  cap drop symptomatic
132  cap label drop symptom
133  /*
134
135  destring temp, replace
136  destring pulse, replace
137  destring rr, replace
138  destring sbp ,replace
139  destring spo2, replace
140  */
141
142  cap drop age_check
143  ge age_check =(dos-dob)/365
144  // Exclusion criteria////
145  cap drop exclude
146  cap lab drop exclude
147
148  gen exclude =0
149
150      replace exclude =3 if age_check <14 // **Under 2s Dropped already
     exceluded by data steward**
151      replace exclude =1 if hx_chf =="Yes"
152      replace exclude =2 if hx_other_lung=="Yes"
153      replace exclude =4 if temp >38.0 & antipyr_home =="Yes" & antipyr_hours
      <4
154      replace exclude =5 if not_being_screened_for_covid =="Yes"
```

```
155
156   lab def exclude 0 "Included" 1"Prior CHF" 2 "Prior Lung disease" 3 "Age <
      14 years" 4 "Confounding use of antypyretic" 5 "Not being screened for
      CoVid 19"
157
158   lab val exclude exclude
159
160
161   // Define abnormal for each then any
162   // define hypoxia
163   cap drop hypoxia
164   cap lab drop hypoxia
165   destring sa02, replace
166   gen hypoxia =1 if sa02 <=92
167    replace hypoxia = 0 if sa02>92  & sa02 <.
168
169   // Define tachycardia
170   cap drop tachycardia
171   cap lab drop tachycardia
172   lab def tachycardia 1 "Tachycardia" 0 "Normal HR"
173
174   gen tachycardia =1 if hr >100 & age_check >=18 & hr !=.
175    replace tachycardia =1 if hr > 105 & age_check >=14 & age_check <18 & hr
      !=.
176    replace tachycardia =1 if hr > 120 & age_check >=5 & age_check <14 & hr !=.
177    replace tachycardia = 0 if tachycardia==.
178
179   lab val tachycardia tachycardia
180
181   //Define hypotension
182   cap drop hypotensive
183   cap lab drop hypotensive
184   lab def hypotensive 1 "Hypotensive" 0 "Normotensive"
185
186   gen hypotensive =1 if sbp <=80 & age_check >18
187    replace hypotensive =1 if sbp <80 & age_check >=14 & age_check<18
188    replace hypotensive = 0 if hypotensive ==.
189   lab val hypotensive hypotensive
190
191   //Define febrile
192   cap drop febrile
193   cap lab drop febrile
194   lab def febrile 0 "Afebrile"  1 "Febrile"
195    gen febrile =1 if temp >=100.4 & temp <.
196    replace febrile =0 if temp <100.4
197   lab val febrile febrile
198
199   //define tachypnea
200
201   cap drop tachypnea
202   cap lab drop tachypnea
203   lab def tachypnea 0 "Normal RR " 1 "Tachypneic"
204
205   gen tachypnea =1 if rr >22 & age_check >=18
206       replace tachypnea =1 if rr > 20 & age_check >=14 & age_check <18
207       replace tachypnea =1 if rr > 30 & age_check>=5 & age_check<14
208       replace tachypnea= 0 if tachypnea==. & rr <.
209   lab val tachypnea tachypnea
```

```
210
211   //
212    cap drop vs_norm
213    cap lab drop vitals
214    lab def vitals  1 "Normal VS" 0 "Abnormal VS"
215    gen vs_norm =1 if febrile+tachycardia +tachypnea +hypotensive + hypoxia
       ==0
216    replace vs_norm =0 if  vs_norm ==. & ((febrile+tachycardia +tachypnea +
       hypotensive + hypoxia)  >0) & ((febrile+tachycardia +tachypnea +hypotensive
        + hypoxia ) <.)
217    lab val vs_norm vitals
218
219   // Ho  That (among symptomatic patients being screened for Covid) Lung
       POCUS is never (<2%) normal.
220
221   // Ha That (among symptomatic patients being screened for Covid) Lung
       POCUS maybe abnormal (= Cw_covid)
222
223   //Actually testing the Null: Among patients with a Lung POCUS c/w Covid
       vital signs are never (<2% of cases normal)
224   // Actual Ha Among patients with a Lung POCUS c/w Covid   vital signs may
       be  abnormal >2% of the time.
225
226   // Variables needed for priamry outcome 1. norm vitals 2. US C/W covid  3.
       Total N of abnormal US These variables are
227   // vs_norm , norm_bin  , cw_covid
228
229   // Vitals_normal
230
231    // US not enterd i dbase - now in dbase but don't want ot start over
232    //replace normal = "No" if csn =="xx"// see original
233   // replace normal = "No" if csn =="xx"//see original
234
235
236
237   // Total number of US to include in N
238   cap drop us_pos_covid
239   gen us_pos_covid =.
240   replace us_pos_covid =1 if regexm(md_covid ,"Yes")
241   replace us_pos_covid =1 if regexm(md_covid ,"Yes")
242   replace us_pos_covid =0 if regexm(md_covid ,"No")
243
244
245   tab us_pos_covid  ,mis
246
247
248   lab var us_pos_covid "N (US c/w CoVid) for bitest"
249
250   // Alternatively for non-immediate form of test
251   bitest us_pos_covid == 0.02 if vs_norm==1&  exclude==0 ,detail
252
253
254   //
255   // Ct pos variable
256   cap drop pos_ct_covid_1
257   gen pos_ct_covid_1 =0
258   replace  pos_ct_covid_1  =1 if regexm(ct_covid , "Yes")
259   diagt pos_ct_covid_1 us_pos_covid  ,sf
```

```
260
261   //
262   //
263   cap drop over_read_us_pos
264   gen  over_read_us_pos  =1 if normal =="No" |regexm(normal, "No - Single
      image only")
265   replace over_read_us_pos =0 if over_read_us_pos ==.
266
267   //cap drop age_check
268   format %td dob
269   //gen age_check  = (dos -dob)/365.25
270   cap drop age1
271
272   /* Figure 1  Initial N will be  _N
273                  Exclusions will be exclude 1-5
274
275   // Table 1
276   //Gender
277   // Age
278   // Day
279   */
280   cap drop antipyretic_lt6
281   gen antipyretic_lt6=0  if antipyr_home =="Yes"
282   replace antipyretic_lt6 =1 if antipyr_hours <=6
283
284
285   //cough dyspnea subj_fever meas_fever duration antipyr_home antipyr_hours
      ausc_clear ausc_crackles aus_wheez_ronc hx_chf hx_other_lung cw_covid
      not_being_screened_for_covid sore_throat fatigue headache myalgias
      diarrhea vomiting exp_known_case
286   cap lab drop
287   lab def present_absent  0 "Absent" 1 "Present"
288
289   foreach var of varlist cough dyspnea subj_fever meas_fever  ausc_clear
      ausc_crackles aus_wheez_ronc hx_chf hx_other_lung  sore_throat fatigue
      headache myalgias diarrhea vomiting cxr_covid ct_covid{
290
291       cap drop `var'_bin
292       gen `var'_bin =1 if `var'=="Yes"
293       replace `var'_bin = 0 if `var' =="No" | `var' ==""
294       lab val `var'_bin present_absent
295   }
296
297
298   cap drop antipyr_home_bin
299   encode antipyr_home ,gen(antipyr_home_bin)
300
301   //label for table  cough_bin dyspnea_bin subj_fever_bin meas_fever_bin
      ausc_clear_bin ausc_crackles_bin aus_wheez_ronc_bin hx_chf_bin
      hx_other_lung_bin sore_throat_bin fatigue_bin headache_bin myalgias_bin
      diarrhea_bin vomiting_bin
302   cap lab drop hypoxia
303   lab def hypoxia  0 "Normoxia" 1 "Hypoxia"
304   lab val hypoxia hypoxia
305
306   lab var cough_bin "Cough"
307   lab var dyspnea_bin "Dyspnea"
308   lab var subj_fever_bin "Subjective fever at home"
```

```
lab var meas_fever_bin "Measured fever at home"
lab var ausc_clear_bin "Lungs clear on auscultation"
lab var ausc_crackles_bin "Crackles/Rales on auscultation"
lab var aus_wheez_ronc_bin "Wheezing or ronchi on auscultation"
lab var sore_throat_bin "Sore throat"
lab var fatigue_bin "Fatigue"
lab var headache_bin "Headache"
lab var myalgias_bin "Myalgias"
lab var diarrhea_bin "Diarrhea"
lab var vomiting_bin "Nausea/vomiting"
lab var hypoxia "Hypoxic"
lab var tachycardia "Tachycardic"
lab var tachypnea "Tachypneic"
lab var hypotensive "Hypotenison"
 table1_mc if exclude  ==0  ,vars(gender  cate\ age_check conts\ duration conts\  subj_fever_bin  cate\  cough_bin  cate\ dyspnea_bin cate\ subj_fever_bin cate\ sore_throat_bin cate\ fatigue_bin cate\  headache_bin  cate\ myalgias_bin cate\ diarrhea_bin cate\ vomiting_bin cate\  vs_norm cate\tachycardia cate\ tachypnea  cate\  hypotensive cate\ hypoxia cate \ ausc_clear_bin  cate\ausc_crackles_bin  cate\ aus_wheez_ronc_bin  cate ) by (us_pos_covid)  total(before) saving(Table_1_covid_by_US.xlsx ,replace)



  table1_mc if exclude  ==0  ,vars(gender  cate\ age conts\ duration  conts\  subj_fever_bin  cate\  cough_bin  cate\ dyspnea_bin cate\subj_fever_bin cate\ sore_throat_bin cate\ fatigue_bin cate\  headache_bin  cate\ myalgias_bin cate\ diarrhea_bin cate\ vomiting_bin cate\ ausc_clear_bin cate\ausc_crackles_bin  cate\ aus_wheez_ronc_bin  cate ) by(us_pos_covid) total(before)

/* Next steps
    1. Compare performance of CXR and Lung POCUS versus CT with CT as the   criterion refenrece(Gold standard)

    2. Compare inter rater reliability between live read and over-read -blinded

    3. Compare inter rater reliabilty between two over readers

*/
 //  1. comparison of modalities

//// Diagt output

cap frame drop sens
frame create sens   modality sen  sen_lb sen_ub spec spc_lb spec_ub ppv ppv_lb ppv_ub npv npv_lb npv_ub lrpos lrpos_lb lrpos_ub lrneg lrneg_lb lrneg_ub  auc auc_lb auc_ub

foreach var of varlist us_pos_covid   cxr_covid_bin ausc_crackles_bin {

 diagt pos_ct_covid_1  `var'  ,sf0

frame post sens (`var') (r(sens)) (r(sens_lb)) (r(sens_ub))  (r(spec)) (r(spec_lb)) (r(spec_ub)) ( r(ppv)) (r(ppv_lb)) (r(ppv_ub)) (r(npv)) (r(npv_lb)) (r(npv_ub))  (r(lrp))  (r(lrp_lb)) (r(lrp_ub)) (r(lrn)) (r(lrn_lb)) (r(
```

```
      lrn_ub))  (r(roc)) (r(roc_lb)) (r(roc_ub))

}

frame sens :save  table2_covid_pull2.dta, replace


 //  Inter rater reliabilty

 //kappaetc pos_ct_covid_1 cxr_covid_bin

 //kappaetc pos_ct_covid_1 cw_covid

 //kappaetc ct_nil_acut_pulm mdUS_normalcopy_bin


 ///
foreach var of varlist excess_long_b excess_short_b thickened_pleura
effusion consolidation atelectasiss normal air_bronchograms
excess_long_bcopy excess_short_bcopy thickened_pleuracopy effusioncopy
consolidationcopy atelectasisscopy normalcopy air_bronchogramscopy {

    cap drop n_`var'
    gen n_`var' =1 if `var' =="Yes"
    replace n_`var' = 0 if `var' =="No"  |`var' ==""



}

foreach var of varlist excess_long_b excess_short_b thickened_pleura
effusion consolidation atelectasiss air_bronchograms  normal{

   di "_____________________________________"
   di "_____________________________________"

   di "`var'"


    kappaetc n_`var'  n_`var'copy

     di "`var'"

}



/* References

     bitest stata manual, Hoel  Mathematicla Statisitics 5Ed Wiley,

     table1   Phil Clayton, ANZDATA Registry, Australia, phil@anzdata.org.au


     diagt  Paul T Seed       (Paul.Seed@kcl.ac.uk)
        Maternal & Fetal Research Unit, GKT School of Medicine, KCL
        North Wing, St Thomas' Hospital, Lambeth Palace Road,
        London SE1 7EH
```

```
403
404       kappaetc  Daniel Klein
405        International Centre for Higher Education Research Kassel
406        Kassel, Germany
407        klein@incher.uni-kassel.de
408
409    */
410
411
412
```
